# A prospective observational cohort pilot study of the association between midazolam use and delirium in elderly endoscopy patients

**DOI:** 10.1186/s12871-021-01275-z

**Published:** 2021-02-16

**Authors:** Dickson Lee, Fiona Petersen, Maurice Wu, Gwenda Chapman, Melanie Hayman, Kerrilyn Tomkins, Jeremy Fernando

**Affiliations:** 1 Rockhampton Hospital, Central Queensland Hospital and Health Service, Canning Street, Rockhampton, QLD 4700 Australia; 2 The University of Queensland Rural Clinical School, 78 Canning St, The Range, QLD 4700 Australia; 3 Central Queensland University, 554-700 Yaamba Rd, Norman Gardens, QLD 4701 Australia; 4 St Vincent’s Hospital, 22-36 Scott St, Toowoomba City, QLD 4350 Australia

**Keywords:** Anaesthesia, Sedation, Midazolam, Delirium, Elderly, Endoscopy

## Abstract

**Background:**

Midazolam is a benzodiazepine commonly used in procedural sedation and general anaesthesia. Current anaesthetic guidelines advise the avoidance of benzodiazepines in elderly patients due to concerns of an increased risk of delirium. Delirium is associated with significant patient morbidity and mortality, while also increasing health costs. Despite this, midazolam is often used in elderly patients undergoing low risk procedures due to the benefits of rapid onset, anxiolysis and haemodynamic stability compared to other sedatives. To date, studies describing the relationship between midazolam use and delirium in elderly patients undergoing low risk procedures, such as endoscopy, are limited.

**Method:**

This was a prospective observational cohort pilot study identifying the prevalence of delirium pre-procedure and incidence of delirium post-procedure in elderly endoscopy patients receiving midazolam. The study population was elderly patients greater than 65 years of age, without underlying cognitive dysfunction, undergoing elective endoscopy. Electronic databases were used for collection of demographic and clinical information. Delirium was identified through the administration of the Family Confusion Assessment Method survey; this was administered to carers of the study population 24–48 h pre and post procedure to categorically identify the presence or absence of delirium.

**Results:**

Fifty-eight participants were recruited for this study and eighteen were subsequently excluded based upon additional exclusion criteria. Forty patients were included in the final results. American Society of Anaesthesiology Classification (ASA) of patients were as follows: 1 (9 patients), 2 (12 Patients), 3 (16 Patients) and 4 (3 patients). Patients underwent gastroscopy, colonoscopy or combined gastroscopy and colonoscopy. This study identified no cases of delirium in elderly patients after administration of midazolam for elective endoscopy procedures 24–48 h post-procedure. Additionally, a high proportion of elderly patients were found to have received midazolam.

**Conclusion:**

No episodes of delirium were identified in this study. This finding runs counter to current guideline recommendations regarding midazolam use in the elderly patient and that elderly patients undergoing elective endoscopy represent a significantly different patient population compared to those previously studied. This study suggests that in the study population that the risk of delirium in patients exposed to midazolam in elective endoscopy was not demonstrated and that it may be safe to perform experimental studies to elucidate the safety of midazolam in larger studies.

## Background

The use of benzodiazepines in anaesthetic practice is well-established. They produce amnesia, anxiolysis and sedation. The time leading up to surgery is often stressful for patients and these medications play an important role in improving patient comfort [1]. The use of benzodiazepines also allows anaesthetists and sedationists to decrease the dose of other medications that cause haemodynamic instability (i.e. Propofol), however, there are studies that show an increased risk of the development of delirium after benzodiazepine exposure [2, 3]. Midazolam is commonly used in elderly patients undergoing endoscopy and the purpose of this study is to elucidate whether this study protocol was feasible and resulted in the detection of delirum in in elderly patients exposed to midazolam undergoing low-risk ambulatory surgery.

Delirium is a serious medical condition defined as the presence of inattention, fluctuating consciousness and disorganisation of thinking. This acute confusional state is known to have significant impacts on morbidity and mortality [4]. A study by Leslie and Inouye (2011) showed that the presence of delirium is associated with a one-year increase in mortality by 62% [5]. In addition, one patient with delirium can cost the health service between $16,303 to $64,421 as a result of increased length of hospitalisation, increased nursing requirements, lasting functional declines, and increased rates of nursing home placement [5].

In the post-operative period, emergence delirium and post-operative delirium exist as separate entities. Emergence delirium is self-limiting, does not fluctuate and only lasts for a short period of time; this is in contrast to post-operative delirium, an acute event in the post-operative period discretely defined in the Diagnostic and Statistical Manual of Mental Disorders (DSM) [6]. While there are multiple etiologies, the incidence of delirium is highest in patients who are elderly or have pre-existing cognitive impairment [7]. Medications with sedative and anticholinergic effects are also commonly implicated in contributing to the development of delirium, and can include benzodiazepines, opioid medications, and anti-depressant medications [8].

A widely cited article by *Marcantonio* et al. (1994) showed that after the administration of benzodiazepines, patients were three times more likely to develop delirium (95% CI; 1.3–6.8). This study further showed that longer acting benzodiazepines and higher doses of benzodiazepines were more strongly associated with the development of delirium [2]. While the association between the development of delirium in patients who have undergone major surgery and critically ill patients in intensive care settings is well established, there is a paucity of data regarding the relationship between short-acting benzodiazepines, such as midazolam, and the development of delirium, in low-risk day surgery patients [9].

As a result of the above paper by *Marcantonio* et al. (1994), anaesthetic guidelines recommend the avoidance of benzodiazepines in elderly patients [2, 10]. Despite this, in an Australian study by *Leslie* et al. (2017), a significant proportion (37.4%) of patients between 18 and 95 years of age in Victorian centres undergoing endoscopy were found to have received midazolam [11].

Midazolam is the shortest acting benzodiazepine and is unique in its chemical structure. Midazolam’s rapid onset is attributable to its direct action and high affinity to benzodiazepine receptors. Its quick offset is a result of rapid oxidation of the methyl group on its imidazole ring; this is in contrast to the slower oxidation of the methylene group on the diazepine ring of classical benzodiazepines [1, 12].

While no studies look at the relationship between midazolam use and delirium as a primary outcome, there are studies that appear to show that midazolam’s effect on delirium is not concordant with the findings of *Marcantonio* et al. In a randomized-control trial studying the incidence of delirium in patients undergoing hip fracture repair, a univariate analysis showed no significant relationship between the dosage of midazolam administered and delirium, with an odds ratio of 0.97 (95% CI; 1.02–1.07) [13]. A second randomized-control trial studied the safety of midazolam in upper endoscopy and demonstrated that cardiopulmonary stability is maintained with midazolam use. Whilst no increase in post-operative cognitive dysfunction is reported, delirium was not measured [14]. As the relationship between midazolam use and delirium in elderly patients undergoing low risk endoscopy is poorly understood, this study has been designed as a step towards improving the evidence behind anaesthetic practice.

## Methods

### Study setting

This study was based at Rockhampton Hospital, the largest hospital in regional Central Queensland, Australia. Male and female patients ≥65 years of age undergoing endoscopic procedures between September 2018 and March 2019 were included in the study.

### Recruitment

Patients were identified through the National Bowel Cancer Screening Program (NBCSP) and General Practitioner referrals for endoscopy through the Rockhampton Hospital General Surgery Clinic. Consent was obtained through return mail or via face-to-face recruitment at the pre-admission clinic prior to procedure. In all instances, patients were asked to nominate a designated carer to participate in the study and consent was obtained from both parties.

### Eligibility criteria

To be eligible for this study, participants were required to be elderly (> 65 years old) patients undergoing elective endoscopic procedures in the Rockhampton region.

Exclusion criteria included; (a) an inability to consent, (b) pre-existing diagnosis of dementia, (c) non-English speaking carers and (d) patients with no contactable carer. Exclusion of patients with a diagnosis of dementia was to reduce the potential for a significant confounder.

There were several groups of patients who were included to the study initially, but subsequently excluded. These groups are as follows: (a) patients who did not receive midazolam, (b) patients did not undergo an endoscopy, (c) patients or carers who withdrew consent or were unable to be contacted within 24 to 48 h pre and post procedure and (d) patients who had their procedure prior to the administration of the pre-procedure interview. The anaesthetists administering sedation were unaware of whether a patient was enrolled in the study.

### Measurement of delirium

In this study, the Family Confusion Assessment Method (FAM-CAM) was used. This tool is validated for delirium screening and categorically determines the presence or absence of delirium. When compared to the original Confusion Assessment Method (CAM), it has been shown to have a high level of agreement (over 95%) [15]. The CAM itself has a sensitivity and specificity to detect delirium of > 90% when validated against psychiatrist diagnosed delirium [16]. Additionally, the FAM-CAM can be administered to any carer (i.e. spouse, family member, friend, etc.) and maintains high fidelity.

**Fig. 1:**
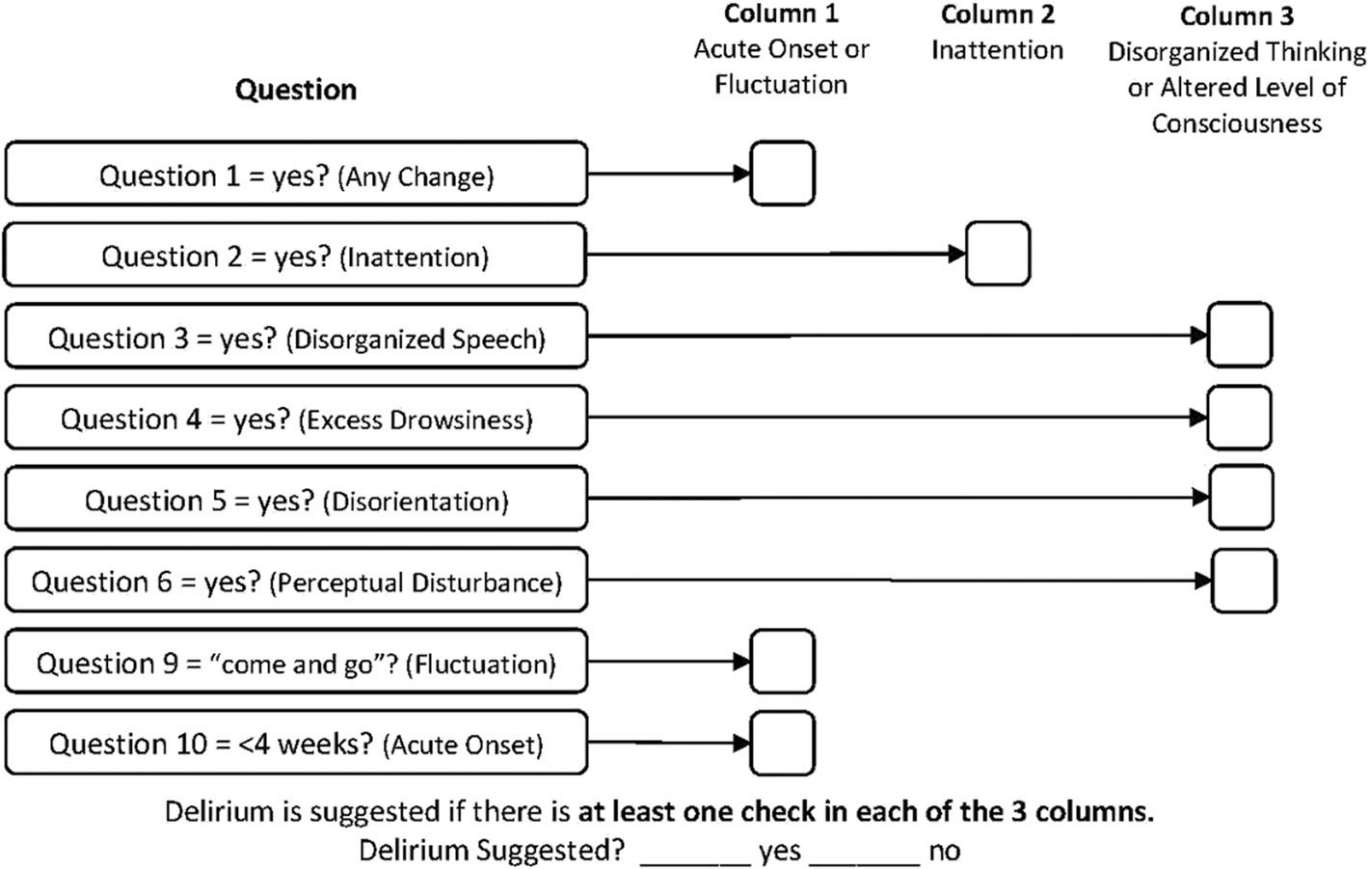
Family confusion assessment method (FAM-CAM) Algorithm. Patients must score at least one criterion in each column to be diagnosed with delirium

The Family Confusion Assessment Method (FAM-CAM) 11 question survey (*Appendix 1*) was administered over the phone to the patients’ carers 24–48 h in advance of their procedure, then 24–48 h after their procedure. By scoring the results of the survey against the 3 required criterion to diagnose delirium, based on the algorithm in Fig. 1, patients were identified to have the presence or absence of delirium.

### Variables

The possible outcomes of study participants include the presence or absence of delirium based upon the FAM-CAM. All participants must have been exposed to midazolam to be included in the study. Potential confounders exist within the study as follows:

1. Co-administered anaesthetic agents
2. Potential for variable depths of anaesthesia
3. Non-standardised midazolam dosing regimens

### Bias

Sources of bias may include the anaesthetists’ awareness of the study occurring, and the potential for subjectivity of nominated carers in providing responses to the FAM-CAM. Anaesthetists were not made aware of which patients were participants of the study and patients did not know whether they would receive midazolam, and were subsequently excluded if midazolam was not administered. Standardised clarifications were provided to nominated carers regarding the FAM-CAM questionnaire to assist in improving understanding and quality of responses. Additionally, there is bias in the exclusion of patients with dementia, however, given the known major impact of dementia on the development of delirium, this was identified as an unacceptable confounder and excluded.

### Data collection

Following consent from the participant and their nominated carer after the procedure, information regarding age, and medical co-morbidities were collected from electronic databases. Following the procedure, the Automated Anaesthetic Record Keeper electronic record was accessed to determine whether midazolam was administered, the dosage administered and concurrently administered medications, as well as, height and weight data. Data not found on the electronic system was obtained via chart review.

### Ethics

Ethical approval was obtained from the Central Queensland Hospital and Health Service Human Research Ethics Committee (HREC/18/QCQ/30). All participants (patient and carer pairs) required informed written consent. All methods were carried out in accordance with local guidelines and regulations.

## Results

**Fig. 2:**
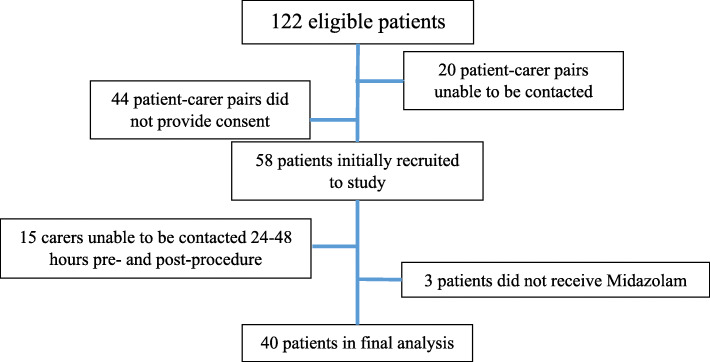
Flowchart of patient selection process

One hundred twenty-two patients met the inclusion criteria and were deemed eligible for the study during the study period of 7 months. Contact was made with 102 patients and consent received from 58 patient and carer pairs. Of the consented participants, 15 patient-carer pairs were excluded from analysis due to their procedures being brought forward or carers being unable to be contacted within 24–48 h of the procedure. A further 3 patient-carer pairs were subsequently excluded as the patients were found not to have been administered midazolam (Fig. 2). There were 22 (51.2%) female patients and 21 (48.8%) male patients. The mean age was 71.9 +/− 4.6 (Range: 65 years old – 85 years old). The average weight of patients was 86.1 kg +/− 22.4 kg (Range: 50 kg – 166 kg). Twenty-nine patients (72.5%) underwent colonoscopies, 5 (12.5%) patients underwent gastroscopies and 9 (22.5%) patients underwent combination colonoscopy and gastroscopy. Demographic data is reported in the Table 1.

**Table 1:** Demographic data regarding patients studied

Patient characteristics	Total Sample ***n*** = 40	
**Demographic**	**n**	**%**
Age (years) (mean (SD))	71.9	4.6
Weight (kg) (mean (SD))	86.1	24.4
**Gender**		
Male	21	52.5%
Female	22	55.0%
**Type of procedure**		
Colonoscopy	27	67.5%
Gastroscopy	5	12.5%
Colonoscopy + gastroscopy	8	20.0%
**Medical Comorbidities**		
No past medical history	9	22.5%
*Cardiovascular*		
Hypertension	23	57.5%
Ischaemic heart disease	4	10.0%
Atrial fibrillation	6	15.0%
Congestive cardiac failure	3	7.5%
Cardiomyopathy	1	2.5%
*Respiratory*		
Asthma	3	7.5%
Chronic obstructive pulmonary disease	5	12.5%
Obstructive sleep apnoea	5	12.5
Gastrointestinal/hepatic		
Chronic liver disease	1	2.5%
Gastroesophageal reflux disease	11	27.5%
*Endocrine*		
Diabetes mellitus	9	22.5%
Hypothyroidism	2	5.0%
*Neurological*		
Cerebrovascular disease	3	7.5%
Migraine	2	5.0%
*Psychological*		
Anxiety	2	5.0%
Depression	6	15.0%
*Other*		
Chronic kidney disease	4	10.0%
Gout	2	5.0%
Glaucoma	4	10.0%
Rheumatoid arthritis	2	5.0%
History of malignancy	6	15.0%

Forty patients received midazolam during their procedure. The dosage of midazolam received ranged from 1.0 mg to 4.0 mg with a mean dose of 1.73 mg +/− 0.7 mg. The mean mg/kg dose of midazolam was 0.021 mg/kg +/− 0.008 mg/kg, the range of mg/kg dosing was 0.012 mg/kg to 0.048 mg/kg. All patients in the study population received Propofol, either as a bolus dose or target controlled infusion. Thirty-seven (92.5%) patients received opioid medications concurrently; 26 (65.0%) patients received fentanyl, whilst 11 (27.5%) patients received alfentanil (Table 2*)*.

**Table 2:** Medications administered intra-operatively

	n	%
**Medications administered intraoperatively**		
Number of patients administered midazolam	40	100%
Midazolam dosing range (mg)	1.00–4.00	
Dose in those administered midazolam (mg) (mean (SD))	1.73	0.7
Dose in those administered midazolam (mg/kg) (mean (SD))	0.021	0.008
**Other medications administered intraoperatively**		
Number of patients administered propofol	40	100%
Number of patients administered fentanyl	26	65%
Number of patients administered alfentanil	11	27.5%
Number of patients not receiving opioids	3	7.5%

No patients in the study population were found to have post-operative delirium 24–48 h after their procedure based on the FAM-CAM screening tool. This result was consistent across patients undergoing gastroscopy, colonoscopy and combined gastroscopy and colonoscopy procedures. There was an absence of postoperative delirium in all patients administered midazolam in the study population.

## Discussion

The aim of the study was to look at the feasbilty of this study protocol and the relationship between midazolam use and the development of delirium in elderly patients ≥65 years of age undergoing low-risk endoscopy procedures. There is currently a paucity of evidence around the use of midazolam in this patient demographic and existing evidence regarding midazolam is likely to inaccurately portray its impact on the development of delirium by studying the benzodiazepine class collectively; including longer acting agents such as diazepam and temazepam. Much of the existing data involves critically ill intensive care patients, or patients undergoing major surgery, which act as further confounders. This study aims to highlight the relationship between midazolam use and delirium as a primary outcome in elderly low risk day surgery patients. Our study, acknowledging its small sample size, found zero cases of delirium. This is in keeping with the hypothesis that the available evidence regarding midazolam exposure and delirium are not reflective of this low risk patient population.

Our study showed a high rate of midazolam administration in elderly patients undergoing low risk endoscopy at the study site [2]. Forty of forty-three (93.0%) elderly patients meeting inclusion criteria received midazolam; this is contrary to guideline recommendations. *Agostini and Inouye (2003),* showed that in those greater than 65 years of age, the incidence of delirium in postoperative patients of all procedure types is 15 to 53% [17].

A study by *Aya* et al *(2019)* studied the incidence of delirium in elderly patients undergoing ambulatory surgery. This study found an incidence of delirium to be 1.4% (2 of 141 patients) three to 5 days after surgery using the FAM-CAM tool. While a larger sample size is needed to validate these results, it is important data that shows a low rate of post-operative delirium after ambulatory surgery; this suggests that patients undergoing ambulatory procedures form a demographic that is significantly different to previously studied groups. The findings of this paper compliment the goals of our study by emphasizing the impact of major surgery on the development of delirium and how ambulatory surgery differs [18].

In this low risk population, there was an absence of postoperative delirium in all patients who received midazolam. This finding is discordant to the results of the study by *Marcantonio* et al. (1994), which showed a significantly increased incidence of delirium following the administration of benzodiazepines and additionally, the study population demonstrated an absence of delirium despite the presence of the previously identified confounding factors making delirium more likely [2]. While the results from this study are promising, there are several factors that limit the conclusions of our study. The authors acknowledge that a significant limitation of the study is a result of the small sample size. The initially hypothesized incidence of delirium in the post-operative elderly population based on currently available research had likely underestimated the required numbers to impart adequate study power. While a larger sample size would significantly improve the power of the study, this was not possible due to (1); a high proportion of patient-carer pairs not providing consent, and (2); factors which did not allow for the FAM-CAM interview to be administered 24–48 h pre and post procedure (i.e. procedures being brought forward). Additionally, the authors acknowledge that the study dropout rate secondary to inability to follow up patient-carer pairs introduces additional bias in the study. Despite these limitations, based upon the Hanley and Lippman-Hand’s rule of threes, it could be suggested that the 95% confidence interval for the incidence of post-operative delirium in midazolam exposed elderly patients undergoing endoscopy is between 0 and 7.5% [19].

A future area of research includes a larger or multi-centre study to validate our findings through the expansion of catchment and sample size. If a larger study with a similar methodology was conducted this may produce equipoise to perform a definitive randomised control trial on this topic.

## Conclusion

This study suggests that a high proportion of elderly patients without underlying cognitive impairment undergoing low risk endoscopy procedures at the study site are receiving midazolam and that there is a low risk of delirium with midazolam exposure in the study population. While the findings of this study are promising, larger or experimental studies are necessary to prove the safety of midazolam in elderly elective endoscopy patients.

## Data Availability

The data and materials collected for the current study are not publically available to maintain patient confidentiality, but may be available from corresponding author upon reasonable request.

## Appendix

**Table 3:** Family Confusion Assessment Method (FAM-CAM) questionnaire administered to participants

**Family Confusion Assessment Method** 1. I’d like you to think about the past [day]. During this [day], have you noticed any changes in his/her thinking or concentration, such as being less attentive, appearing confused or disoriented (not knowing where he/she was), behaving inappropriately, or being extremely sleepy all day? 2. Did he/she have difficulty focusing attention, for example, being easily distracted or having trouble keeping track of what you were saying at any time 3. Was his/her speech disorganized, incoherent, rambling, unclear, or illogical at any time? 4. Did he/she seem excessively drowsy or sleepy during the daytime at any time? 5. Was he/she disoriented, for example, thinking he/she was somewhere other than where he/she was, or misjudging the time of day at any time? 6. Did he/she seem to see or hear things which weren’t actually present, or seem to mistake what he/she saw or heard for something else at any time? 7. Did he/she behave inappropriately, such as wandering, yelling out or being combative or agitated at any time? 8. Please tell us more about the changes you noticed in any of the behaviours in #1–7 9. Were any of the changes present all the time, or did they come and go from day to day? 10. When did these changes first begin? 11. Overall, have these changes been getting better, worse, or staying the same?	

## Abbreviations

ASA: American Society of Anesthesiology
DSM: Diagnostic and Statistical Manual of Mental Disorders
NBCSP: National Bowel Cancer Screening Program
HREC: Human Research Ethics Committee
FAM-CAM: Family Confusion Assessment Method
CAM: Confusion Assessment Method
AARK: Automated Anaesthetic Record Keeper

## References

[1] Griffin CE, Kaye AM, Bueno FR, Kaye AD (2013). Benzodiazepine pharmacology and central nervous system-mediated effects. Ochsner J [Internet].

[2] Marcantonio ER, Juarez G, Goldman L, Mangione CM, Ludwig LE, Lind L (1994). Lee TH. The relationship of postoperative delirium with psychoactive medications. JAMA..

[3] Frölich MA, Arabshahi A, Katholi C, Prasain J, Barnes S (2011). Hemodynamic characteristics of midazolam, propofol, and dexmedetomidine in healthy volunteers. J Clin Anesth.

[4] Silverstei (2013). Postoperative delirium and cognitive dysfunction. J Dalian Med Univ.

[5] Leslie DL, Inouye SK. The importance of delirium: Economic and societal costs. J Am Geriatr Soc. 2011;59(SUPPL. 2):241–43. 10.1111/j.1532-5415.2011.03671.x PMC3415302 22091567

[6] Evered L, Silbert B, Knopman DS, Scott DA, DeKosky ST, Rasmussen LS (2018). Recommendations for the nomenclature of cognitive change associated with Anaesthesia and Surgery-20181. J Alzheimers Dis [Internet].

[7] Radtke FM, Franck M, Hagemann L, Seeling M, Wernecke KD, Spies CD (2010). Risk factors for inadequate emergence after anesthesia: emergence delirium and hypoactive emergence. Minerva Anestesiol.

[8] Boustani MA, Matthews FE, Richardson K, Coulton S, Katona C, Maidment ID (2011). Anticholinergic medication use and cognitive impairment in the older population: the Medical Research Council cognitive function and ageing study. J Am Geriatr Soc [Internet].

[9] Raats JW, Steunenberg SL, Crolla RM, Wijsman JH, te Slaa A, van der Laan L (2015). Postoperative delirium in elderly after elective and acute colorectal surgery: a prospective cohort study. Int J Surg.

[10] Brown C, Deiner S (2016). Perioperative cognitive protection. Br J Anaesth.

[11] Leslie K, Allen ML, Hessian EC, Peyton PJ, Kasza J, Courtney A (2017). Safety of sedation for gastrointestinal endoscopy in a group of university-affiliated hospitals: A prospective cohort study. Br J Anaesth [Internet].

[12] Gerecke M (1983). Chemical structure and properties of midazolam compared with other benzodiazepines. Br J Clin Pharmacol.

[13] Sieber FE, Zakriya KJ, Gottschalk A, Blute MR, Lee HB, Rosenberg PB (2010). Sedation depth during spinal anesthesia and the development of. Mayo Clin Proc [Internet].

[14] Christe C, Janssens JP, Armenian B, Herrmann F, Vogt N (2000). Midazolam sedation for upper gastrointestinal endoscopy in older persons: a randomized, double-blind, placebo-controlled study. J Am Geriatr Soc.

[15] Steis MR, Evans L, Hirschman KB, Hanlon A, Fick DM, Flanagan N (2012). Screening for delirium using family caregivers: convergent validity of the family confusion assessment method and interviewer-rated confusion assessment method. J Am Geriatr Soc.

[16] Inouye SK, Van Dyck CH, Alessi CA, Balkin S, Siegal AP, Horwitz RI (1990). Clarifying confusion: the confusion assessment method: a new method for detection of delirium. Ann Intern Med.

[17] Agostini JV, Inouye SK (2003). Delirium. Principles of geriatric medicine and gerontology.

[18] Aya AGM, Pouchain PH, Thomas H, Ripart J, Cuvillon P (2019). Incidence of postoperative delirium in elderly ambulatory patients: a prospective evaluation using the FAM-CAM instrument. J Clin Anesth.

[19] Hanley JA, Lippman-Hand A (1983). If nothing goes wrong, is everything all right? Interpreting zero numerators. J Am Med Assoc.

